# Tuberculosis incidence, deaths and disability-adjusted life years in children and adolescence, 1990–2021: Results from the Global Burden of Disease Study 2021

**DOI:** 10.1371/journal.pone.0317880

**Published:** 2025-03-10

**Authors:** Xue Li, Yuanyuan Li, Liping Guo, Yongyan Chen, Gaobiao Wang, Hanjuan Zhang

**Affiliations:** 1 Department of Pharmacy, The 7th People’s Hospital of Zhengzhou, Zhengzhou, China; 2 Henan Key Laboratory of Cardiac Remodeling and Transplantation, The 7th People’s Hospital of Zhengzhou, Zhengzhou, China; 3 Department of Pharmacy, Ruijin hospital Affiliated to Shanghai Jiao Tong University School of Medicine, Shanghai, China; 4 Department of Pharmacy, People’s Hospital of Puyang, Puyang, China; Kerman University of Medical Sciences, IRAN, ISLAMIC REPUBLIC OF

## Abstract

**Background:**

For a long time, eliminating tuberculosis (TB) has been an enormous challenge in global health. We aim to use the 2021 Global Burden of Disease Study (GBD 2021) to systematically analyze the TB burden in children and adolescents.

**Methods:**

We used the 2021 GBD to retrieve TB incidence, mortality, and disability-adjusted life years (DALYs) data among people aged 0 to19 years in 204 countries and regions between 1990 to 2021. The data are reported as counts and ratios per 100,000 people and are analyzed by age, sex, location and socio-demographic index (SDI). To quantify the uncertainty of the estimations, we include 95% confidence interval (CI) for each indicator.

**Results:**

Globally, the TB burden among people under the age of 20 has decreased significantly between 2019 and 2021. The overall incidence of TB among children and adolescents fell by 37.4%, from 2.21(95% UI:1.71–2.76) million in 1990 to 1.38(95% UI:1.06–1.76) million in 2021. Sub-Saharan Africa had the highest increase in TB incidence (302.88, 95% UI: 227.85-385.33). The age-standardized incidence rate (ASIR), age-standardized mortality rate (ASMR) and age-standardized DALYs rate (ASDR) of TB in females decreased faster than males. The incidence was higher in children under 5 years old and adolescents aged 15 to 19, showing a bimodal pattern. In addition, mortality caused by multidrug-resistant tuberculosis (MDR-TB) and extensively drug-resistant tuberculosis (XDR-TB) have increased dramatically in several areas. The mortality rates for MDR-TB and XDR-TB in Eastern Europe are 0.04(95% UI:0.02-0.05) and 0.02(95% UI:0.01-0.03) respectively.

**Conclusion:**

Although the burden of TB in children and adolescents has decreased globally, the disease remains a major public health concern, especially in countries with low SDI. To accomplish the ultimate aim of TB elimination, we should continue to invest in TB prevention and control, expand health-care infrastructure construction, and advance TB diagnostic, preventive, and treatment technologies.

## Introduction

TB caused by Mycobacterium tuberculosis, is a chronic infectious disease that primarily affects the lungs, but can also arise in extrapulmonary organs. The principal mode of transmission is via the respiratory system. Most healthy people may establish an efficient immune response to eradicate the virus after initial contact; nevertheless, certain susceptible groups may develop active TB disease [1]. Despite being a persistent, low-level endemic illness over the years, TB remains one of the major causes of death globally and continues to offer major challenges to global eradication attempts [2].

The World Health Organization (WHO) estimates that 10.6 million individuals worldwide affected TB in 2021, with around 1.2 million of them children. Approximately 220,000 children died from the disease that same year [3]. Children represent about 11% of the global TB burden, while they account for 14% of TB-related mortality. Adolescence is increasingly recognized as a key period for tuberculosis infection, disease development, and unfavorable outcomes, however the underlying causes are not fully understood changes in social contact situations and immunological function may be contributing factors [4,5]. Previous research has also indicated a sharp increase in TB incidence during adolescence in high-transmission areas [6]. Consequently, the diagnosis and treatment of TB in children and adolescents are crucial for preventing disease progression and halting the spread of TB. These interventions are essential for reducing the TB burden in these vulnerable populations.

For more than 30 years, the Global Burden of Diseases, Injuries, and Risk Factors Study (GBD) has systematically collected and evaluated estimates of the global incidence and mortality of diseases and injuries, stratified by location, time period, sex and age group [7]. Compared to prior incarnations, GBD 2021 provides a more detailed assessment of the disease burden, focusing on the impact of the COVID-19 pandemic [8]. One of the most significant and immediate effects has been a substantial decline in the number of newly diagnosed TB cases globally, dropping from 7.1 million in 2019 to 5.1 million in 2020. As a result, accurately identifying and measuring TB incidence and mortality is critical for reducing the disease burden among children and adolescents. These efforts are also essential for accelerating the implementation of the WHO’s End TB Strategy [9].

Several studies have investigated the prevalence of TB among children and adolescents. Shang and colleagues utilized GBD 2019 data to report the burden of tuberculosis among adolescents aged 10-24 years at global, national, and regional levels from 1990 to 2019[10]. Lv et al. conducted a study estimating TB burden across eight high-burden countries worldwide [11]. Another study examined global trends in TB across five age groups from 2015 to 2020[12]. Additionally, a study estimated the global burden of all infectious diseases among children and adolescents aged 0-24 years [13]. Although prior studies provided valuable data on TB burden among children and adolescents, most are limited to specific age groups or regions and do not encompass the latest global data. Therefore, in comparison with previous studies, this study analyzes the most recent GBD 2021 data to systematically evaluate the incidence, mortality, and DALYs burden of tuberculosis in people aged 0 to 19 years from 1990 to 2021. The research study is worldwide in scope, encompassing 204 countries and territories and stratified by age, sex, and SDI. Our goal is to provide the most up-to-date and reliable scientific data inform healthcare decision-making and policy implementation, thereby addressing the global public health challenge posed by TB more effectively.

## Methods

### Ethical statement

As this study used publicly available summary data from the Global Health Data Exchange, ethical approval was not required.

### Data sources

The GBD 2021 project estimated the incidence, mortality, and DALYs associated with 371 diseases and injuries for 204 countries and territories from 1990 to 2021. Data sources were identified from vital registration, verbal autopsy, registry, survey, police, or surveillance data across all countries and territories [8]. The general methodologies, primary data sources, and key changes compared to previous iterations of the GBD have been published [14,15]. Utilizing the Global Health Data Exchange (https://vizhub.healthdata.org/gbd-results/), we gathered precise data on the incidence, mortality, and DALYs associated with TB among children and adolescents.

### Case definition

In GBD study, TB is defined as an infectious disease caused by Mycobacterium tuberculosis. This definition includes all types of TB, including both bacteriologically confirmed and clinically diagnosed cases of pulmonary and extrapulmonary TB. According to the 10th revision of the International Classification of Diseases (ICD-10), the coding for TB includes A10-A19.9, B90-B90.9, K67.3, K93.0, M49.0, N74.1, P37.0, U84.3. This study includes three types of TB: 1. Drug-susceptible tuberculosis (DS-TB): TB that is susceptible to isoniazid and rifampicin. 2. Multidrug-resistant tuberculosis without extensive drug resistance (MDR-TB): TB that is resistant to the two most effective first-line antituberculosis drugs (isoniazid and rifampicin) but is not resistant to any fluoroquinolone and any second-line injectable drugs (amikacin, kanamycin, or capreomycin). 3. Extensively drug-resistant tuberculosis (XDR-TB): TB that is resistant to isoniazid and rifampicin, as well as any fluoroquinolone and any second-line injectable drugs [16,17].

Adolescence is the period between childhood and adulthood, with WHO defining it as 10-19 years old [18]. This study focuses on the methodology and statistical analyses used in the GBD 2021 study to estimate the burden of TB among children aged 0 to 9 years and adolescents aged 10 to 19. To have a deeper understanding, we divided the age groups into four subcategories: < 5 years, 5-9 years, 10-14 years, and 15-19 years.

The SDI is a composite indicator of a country’s development level that ranges between 0 and 1. It assesses per capita income, average educational attainment in the population aged 15 and up, and total fertility rate in the population under 25 years. The SDI is divided into quintiles based on 2021 national-level SDI estimates, ranging from low to high: low SDI, low-middle SDI, middle SDI, high-middle SDI, and high SDI [19].

### Statistical analysis

Data were reported as counts and rates per 100,000 population, including incidence, mortality, and DALYs, and were analyzed by age, sex, location, and SDI. TB incidence was estimated using a random-effects meta-analysis approach, employing the DisMod-MR model to integrate epidemiological data. Mortality rates were estimated using the cause-of-death ensemble modeling (CODEm). Final point estimates are presented with 95% uncertainty intervals (UIs), generated using the 2.5th and 97.5th percentiles from a 1000-draw distribution for each metric [20].

We employed age-standardized rates (ASR) and estimated annual percentage change (EAPC) to quantify trends in tuberculosis incidence and mortality among children and adolescents across different regions. EAPC is a widely used summary measure of ASR trends over a specific period [21]. A regression line was fitted to the natural logarithm of the ASR, i.e., y=α+βx+ε ,where y =  $\ln\left(ASR\right)$, and x =  the calendar year. EAPC was calculated as $100\times \left(\text{exp}\left(\text{β}\right)-1\right)$, with the 95% CI reflecting the time trend of the ASR. An increasing trend in ASR was indicated when both the EAPC and the lower bound of the 95% CI were positive; conversely, a decreasing trend was indicated when both the EAPC and the upper bound of the 95% CI were negative.

Pearson correlation analysis was performed to evaluate the relationships between ASIR, ASMR, and ASDR and SDI across 204 countries and territories, aiming to identify potential factors influencing the tuberculosis burden. All statistical analyses were conducted using R software version 4.2.3. Two-tailed tests were used for all statistical assessments, and the significance level was set at *P* <  0.05.

## Results

### Global trend

**Fig 1:**
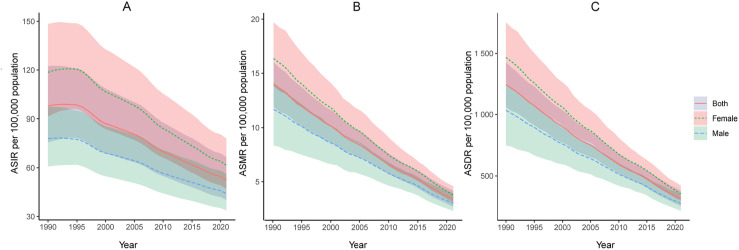
Temporal trends of tuberculosis ASIR **(A), ASMR** **(B), ASDR (C) per 100,000 population by sex, 1990–2021.** ASIR, age-standardized incidence rate; ASMR, age-Standardized mortality rate; ASDR, age-standardized DALYs rate; DALYs, disability-adjusted life years.

Globally, there has been a substantial reduction in the global burden of TB among individuals under the age of 20 between 2019 and 2021. The ASIR, ASMR, and ASDR all decreased progressively, with ASIR falling at a slower rate than ASMR and ASDR. Meanwhile, males showed lower ASIR, ASMR, and ASDR for TB than females (Fig 1). The total TB incident number in children and adolescence decreased by 37.4%, from 2.21(95% UI:1.71–2.76) million in 1990 to 1.38(95% UI:1.06–1.76) million in 2021, while the ASIR per 100,000 population fell from 97.85 (95% UI:75.75–122.38) to 52.52 (95% UI: 40.22–66.60), a 46.3% reduction. The EAPC for ASIR were -2.16 (95% CI: -2.28 to -2.04) (Table 1). The number of deaths decreased by 71.7% from 314,807 (95% UI: 268,570–361,819) in 1990 to 89,086 (95% UI: 70,849–109,974) in 2021, with the ASMR per 100,000 population dropping from 13.94 (95% UI: 11.89–16.02) to 3.38 (95% UI: 2.69–4.17), a 75.8% reduction. The EAPC for ASMR were -3.90 (95% CI: -4.07 to -3.73) (Table 2). The number of DALYs decreased from 28.10 million (95% UI: 23.92–32.24) in 1990 to 8.26 million (95% UI: 6.70–9.99) in 2021, reflecting a 70.6% reduction. Similarly, the ASDR per 100,000 population decreased by 74.8% from 1244.33 (95% UI: 1059.09–1427.44) to 313.24 (95% UI: 254.26–379.00). The EAPC for ASDR were -4.21 (95% CI: -4.40 to -4.01) (S1 Table).

**Table 1:** Incidence, ASIR, and Relative change of Tuberculosis in children and Adolescence in global and 21 regions, with EAPC from 1990 and 2021.

Characteristic	Incidence(95% UI)				Percentage change 1990-2021		1990-2021EAPC of ASIR (95%CI)
	1990		2021				
	Incident cases, No. × 10^3^	ASIR per 100,000	Incident cases, No. × 10^3^	ASIR per 100,000	Incident cases, %	ASIR, %	
Global	2209.89(1710.76-2763.98)	97.85(75.75-122.38)	1384.42(1060.18-1755.37)	52.52(40.22-66.60)	-37.40%	-46.30%	-2.16(-2.28--2.04)
21 Regions							
Andean Latin America	21.88(16.35-28.03)	115.43(86.27-147.87)	6.68(5.02-8.69)	28.23(21.22-36.72)	-69.50%	-75.50%	-4.96(-5.34--4.59)
Australasia	0.28(0.20-0.37)	4.40(3.21-5.89)	0.18(0.13-0.23)	2.38(1.77-3.11)	-35.10%	-46.00%	-2.00(-2.15--1.86)
Caribbean	6.68(5.30-8.15)	44.21(35.12-53.95)	3.71(2.85-4.70)	24.30(18.68-30.80)	-44.40%	-45.00%	-2.04(-2.17--1.92)
Central Asia	13.82(10.56-17.45)	43.76(33.43-55.26)	6.93(5.38-8.67)	20.00(15.53-25.03)	-49.90%	-54.30%	-2.17(-2.70--1.65)
Central Europe	5.29(3.95-6.77)	13.47(10.07-17.24)	1.19(0.90-1.54)	5.05(3.80-6.54)	-77.50%	-62.50%	-3.11(-3.31--2.92)
Central Latin America	13.53(10.70-16.49)	16.37(12.95-19.95)	5.81(4.38-7.63)	6.81(5.14-8.94)	-57.10%	-58.40%	-3.21(-3.45--2.98)
Central Sub-Saharan Africa	94.01(75.81-115.07)	303.38(244.65-371.33)	121.34(96.89-150.21)	164.95(131.71-204.20)	29.10%	-45.60%	-1.91(-2.21--1.61)
East Asia	302.50(232.25-385.97)	65.74(50.48-83.88)	55.57(42.2707-70.11)	16.11(12.25-20.32)	-81.60%	-75.50%	-4.40(-4.57--4.22)
Eastern Europe	23.13(16.66-31.20)	34.39(24.76-46.38)	6.74(4.71-9.19)	14.60(10.21-19.90)	-70.90%	-57.50%	-2.28(-3.00--1.55)
Eastern Sub-Saharan Africa	231.43(187.47-278.33)	208.69(169.05-250.98)	241.71(183.25-308.81)	106.21(80.52-135.69)	4.40%	-49.10%	-2.52(-2.72--2.32)
High-income Asia Pacific	6.30(5.02-7.68)	12.51(9.97-15.27)	0.85(0.59-1.17)	2.76(1.91-3.79)	-86.50%	-78.00%	-4.12(-4.43--3.81)
High-income North America	0.91(0.64-1.23)	1.11(0.79-1.50)	0.94(0.68-1.23)	1.05(0.76-1.37)	3.50%	-5.60%	-0.03(-0.23-0.17)
North Africa and Middle East	76.53(60.34-95.54)	43.29(34.13-54.05)	34.68(26.31-44.04)	14.67(11.12-18.62)	-54.70%	-66.10%	-3.36(-3.58--3.13)
Oceania	2.24(1.73-2.79)	66.57(51.43-82.89)	3.46(2.67-4.31)	54.16(41.86-67.56)	54.40%	-18.60%	-0.58(-0.70--0.45)
South Asia	806.71(589.57-1061.85)	148.72(108.69-195.76)	431.35(318.68-562.10)	63.11(46.63-82.24)	-46.50%	-57.60%	-3.27(-3.45--3.10)
Southeast Asia	284.09(222.08-344.40)	129.19(100.99-156.62)	169.11(132.25-209.60)	73.76(57.68-91.42)	-40.50%	-42.90%	-2.01(-2.24--1.78)
Southern Latin America	2.75(2.05-3.64)	14.18(10.57-18.79)	1.58(1.16-2.07)	8.11(5.93-10.60)	-42.40%	-42.80%	-1.88(-2.07--1.68)
Southern Sub-Saharan Africa	100.16(79.26-122.88)	378.51(299.53-464.37)	94.70(71.24-120.47)	302.88(227.85-385.33)	-5.50%	-20.00%	-0.29(-0.68-0.10)
Tropical Latin America	14.05(10.43-18.52)	20.29(15.06-26.74)	6.91(4.97-9.35)	10.37(7.46-14.03)	-50.90%	-48.90%	-3.00(-3.43--2.56)
Western Europe	7.52(5.40-10.02)	7.65(5.49-10.19)	3.60(2.58-4.85)	3.93(2.81-5.29)	-52.10%	-48.70%	-1.83(-1.96--1.69)
Western Sub-Saharan Africa	196.11(160.81-235.08)	182.43(149.60-218.69)	187.41(147.79-230.62)	69.78(55.03-85.87)	-4.40%	-61.80%	-3.11(-3.41--2.80)
Sex							
Female	1305.91(1006.94-1634.07)	118.59 (91.44-148.39)	785.86 (600.43-995.19)	61.52 (47.01-77.91)	-39.82%	-48.12%	0.78 (-6.59-8.74)
Male	903.98(703.82-1125.30)	78.11(60.81-97.23)	598.57 (457.61-762.53)	44.06 (33.69-56.13)	-33.79%	-43.59%	0.88 (-6.45-8.79)
Age							
<5 years	684.50 (528.18-867.47)	110.41 (85.20-139.93)	342.72 (266.65-432.59)	52.07 (40.51-65.73)	-49.93%	-52.84%	0.08 (-6.92-7.60)
5-9 years	323.16(207.54-485.45)	55.38 (35.57-83.19)	161.79(103.81-244.59)	23.55 (15.11-35.60)	-49.94%	-57.48%	0.38 (-6.60-7.89)
10-14 years	420.74(263.40-604.68)	78.54 (49.17-112.88)	254792.76 (158.01-374.72)	38.22 (23.70-56.21)	-39.44%	-51.34%	0.51 (-6.46-8.01)
15-19 years	781.50(496.30-1137.52)	150.45 (95.55-219.00)	625.12(415.06-897.53)	100.18 (66.52-143.84)	-20.01%	-33.41%	1.56 (-5.43-9.08)
SDI							
Low	524.08(420.88-638.48)	187.45 (150.54-228.37)	515.26(401.66-644.09)	88.20 (68.75-110.25)	-1.68%	-52.95%	2.77 (-4.69-10.82)
Low-middle	864.13(653.14-1096.40)	146.21 (110.51-185.51)	490.10(367.18-630.03)	64.12 (48.04-82.42)	-43.28%	-56.15%	2.27 (-5.11-10.22)
Middle	634.77(494.48-803.49)	83.02 (64.68-105.09)	330.14(253.25-415.15)	44.07 (33.80-55.41)	-47.99%	-46.92%	-0.38 (-7.28-7.04)
High-middle	161.89(122.98-206.46)	43.73 (33.22-55.78)	40.34(30.41-51.80)	13.30 (10.02-17.08)	-75.08%	-69.59%	-1.40 (-7.39-4.98)
High	23.95(18.64-30.01)	9.53 (7.42-11.94)	7.80(5.70-10.27)	3.35 (2.45-4.41)	-67.45%	-64.84%	-2.10 (-8.30-4.52)

Abbreviations: No., number; UI, uncertainty interval; ASIR, age-standardized incidence rate; EAPC, estimated annual percentage change; CI, confidential interval.

**Table 2:** Death, ASMR, and Relative change of Tuberculosis in children and Adolescence in global and 21 regions, with EAPC from 1990 and 2021.

Characteristic	Death (95% UI)				Percentage change 1990-2021		1990-2021EAPC of ASMR (95%CI)
	1990		2021				
	Death cases, No. × 10^3^	ASMR per 100,000	Death cases, No. × 10^3^	ASMR per 100,000	Death cases, %	ASMR, %	
Global	314.81(268.57-361.82)	13.94(11.89-16.02)	89.09(70.85-109.97)	3.38(2.69-4.17)	-71.70%	-75.80%	-3.90(-4.07--3.73)
21 Regions							
Andean Latin America	3.10(2.57-3.62)	16.34(13.56-19.11)	0.23(0.19-0.29)	0.99(0.80-1.23)	-92.50%	-94.00%	-8.68(-8.93--8.43)
Australasia	0.00(0.00-0.00)	0.03(0.03-0.03)	0.00(0.00-0.00)	0.00(0.00-0.00)	-83.40%	-86.20%	-5.80(-6.03--5.56)
Caribbean	1.14(0.92-1.55)	7.57(6.10-10.24)	0.33(0.23-0.57)	2.18(1.48-3.71)	-70.90%	-71.20%	-3.87(-4.13--3.60)
Central Asia	1.58(1.42-1.74)	4.99(4.51-5.52)	0.39(0.32-0.48)	1.11(0.92-1.38)	-75.50%	-77.60%	-4.17(-4.51--3.82)
Central Europe	0.17(0.16-0.18)	0.44(0.41-0.47)	0.01(0.01-0.02)	0.06(0.05-0.07)	-91.80%	-86.30%	-6.25(-6.48--6.02)
Central Latin America	1.76(1.66-1.87)	2.13(2.01-2.26)	0.19(0.16-0.22)	0.22(0.18-0.26)	-89.30%	-89.70%	-7.88(-8.27--7.50)
Central Sub-Saharan Africa	23.20(16.95-30.25)	74.86(54.70-97.60)	10.35(6.77-16.38)	14.07(9.20-22.27)	-55.40%	-81.20%	-3.75(-4.25--3.25)
East Asia	24.81(21.21-28.96)	5.39(4.61-6.29)	0.62(0.52-0.76)	0.18(0.15-0.22)	-97.50%	-96.70%	-8.98(-9.43--8.52)
Eastern Europe	0.36(0.35-0.38)	0.54(0.52-0.56)	0.05(0.05-0.05)	0.11(0.10-0.12)	-86.40%	-80.20%	-3.87(-4.73--3.01)
Eastern Sub-Saharan Africa	59.40(46.94-72.03)	53.57(42.32-64.95)	19.63(14.51-25.72)	8.62(6.38-11.30)	-67.00%	-83.90%	-5.04(-5.27--4.81)
High-income Asia Pacific	0.30(0.25-0.36)	0.60(0.51-0.71)	0.01(0.01-0.01)	0.02(0.02-0.02)	-98.20%	-97.00%	-11.28(-11.59--10.98)
High-income North America	0.03(0.03-0.03)	0.04(0.04-0.04)	0.01(0.01-0.01)	0.01(0.01-0.01)	-82.80%	-84.30%	-6.28(-6.68--5.88)
North Africa and Middle East	8.51(6.76-10.88)	4.82(3.83-6.16)	2.16(1.68-2.89)	0.91(0.71-1.22)	-74.60%	-81.00%	-5.37(-5.58--5.15)
Oceania	0.33(0.23-0.49)	9.68(6.92-14.43)	0.35(0.24-0.49)	5.47(3.78-7.64)	7.30%	-43.40%	-1.44(-1.64--1.25)
South Asia	110.08(90.82-130.71)	20.29(16.74-24.10)	23.31(19.82-26.70)	3.41(2.90-3.91)	-78.80%	-83.20%	-5.35(-5.48--5.22)
Southeast Asia	38.09(30.24-44.01)	17.32(13.75-20.02)	6.69(5.64-7.77)	2.92(2.46-3.39)	-82.40%	-83.10%	-5.44(-5.53--5.35)
Southern Latin America	0.20(0.18-0.21)	1.00(0.95-1.07)	0.03(0.02-0.03)	0.14(0.12-0.16)	-86.10%	-86.20%	-6.72(-7.10--6.33)
Southern Sub-Saharan Africa	5.19(4.29-6.73)	19.63(16.22-25.42)	3.21(2.56-3.97)	10.28(8.18-12.70)	-38.20%	-47.70%	-2.37(-2.81--1.93)
Tropical Latin America	1.33(1.19-1.49)	1.91(1.72-2.15)	0.17(0.14-0.20)	0.25(0.21-0.29)	-87.30%	-86.80%	-7.17(-7.41--6.93)
Western Europe	0.05(0.05-0.05)	0.05(0.05-0.05)	0.01(0.01-0.01)	0.01(0.01-0.01)	-88.60%	-87.80%	-6.84(-6.96--6.73)
Western Sub-Saharan Africa	35.18(26.31-44.59)	32.73(24.48-41.48)	21.35(14.10-32.47)	7.95(5.25-12.09)	-39.30%	-75.70%	-3.44(-3.79--3.10)
Sex							
Female	180.00 (152.10-216.92)	16.35 (13.81-19.70)	89.09(70.85-109.97)	3.78 (3.09-4.57)	-73.19%	-76.89%	-2.84(-7.74-2.31)
Male	134.81(96.67-163.77)	11.65 (8.35-14.15)	40.83(31.25-53.05)	3.01 (2.30-3.91)	-69.71%	-74.20%	-2.50 (-7.41-2.67)
Age							
<5 years	227.74(189.33-264.33)	36.74 (30.54-42.64)	55.17(39.89-71.63)	8.38 (6.06-10.88)	-75.78%	-77.18%	-3.09 (-7.75-1.80)
5-9 years	28.12(22.68-33.39)	4.82 (3.89-5.72)	7.89(6.53-9.44)	1.15 (0.95-1.37)	-71.95%	-76.18%	-2.70 (-7.33-2.17)
10-14 years	19.42(16.59-22.60)	3.63 (3.10-4.22)	7.60(6.61-8.56)	1.14 (0.99-1.28)	-60.85%	-68.54%	-1.76 (-6.41-3.12)
15-19 years	39.52(34.41-44.39)	7.61 (6.62-8.55)	18.43(16.47-20.74)	2.95 (2.64-3.32)	-53.37%	-61.18%	-1.23 (-5.88-3.66)
SDI							
Low	121.22(98.07-143.45)	43.36 (35.08-51.31)	50.73(36.47-66.46)	8.68 (6.24-11.38)	-58.15%	-79.97%	-2.37 (-7.06-2.55)
Low-middle	124.39(102.99-146.76)	21.05 (17.43-24.83)	28.92(24.23-33.71)	3.78 (3.17-4.41)	-76.75%	-82.02%	-2.68 (-7.39-2.27)
Middle	59.69(53.48-65.76)	7.81 (6.99-8.60)	8.73(7.59-10.21)	1.17 (1.01-1.36)	-85.37%	-85.07%	-4.69 (-9.17-0.02)
High-middle	8.53(7.29-9.67)	2.30 (1.97-2.61)	0.61(0.53-0.70)	0.20 (0.17-0.23)	-92.89%	-91.33%	-5.76 (-9.76--1.57)
High	0.82(0.71-0.95)	0.33 (0.28-0.38)	0.04(0.03-0.04)	0.02 (0.02-0.02)	-94.91%	-94.50%	-8.53 (-12.41--4.47)

Abbreviations: No., number; UI, uncertainty interval; ASMR, age-standardized mortality rate; EAPC, estimated annual percentage change; CI, confidential interval.

In females, the ASIR decreased by 48.12%, with an EAPC of 0.78 (95% CI: -6.59 to 8.74). Similarly, males experienced a reduction in ASIR by 43.59%, with an EAPC of 0.88 (95% CI: -6.45 to 8.79) (Table 1). The pattern for mortality and DALYs was similar, with the ASMR and ASDR showing a reduction of 76.89% and 75.83% for females, and 74.20% and 73.43% for males, respectively (Table 2, S1 Table).

In 2021, the incidence for individuals aged less than 5 years, 5-9 years, 10-14 years, and 15-19 years were decreased by 52.84%, 57.48%, 51.34% and 33.41%, respectively. The group aged less than 5 years had the greatest decline in mortality [EAPC = -3.09 (95% CI: -7.75 to 1.80)] and in DALYs [EAPC = -0.02 (95% CI: -6.94 to 7.41)] (Table 2, S1 Table).

TB incidence, mortality, and DALYs varied significantly across regions stratified by SDI. Regions with a High-SDI showed a substantial decline in TB incidence, with a 67.45% and the lowest ASIR of 3.35 per 100,000 people in 2021. In contrast, regions with a low-SDI experienced only a modest reduction of 1.68%, maintaining a considerably higher ASIR of 88.02 per 100,000 in 2021(Table 1).

**Fig 2:**
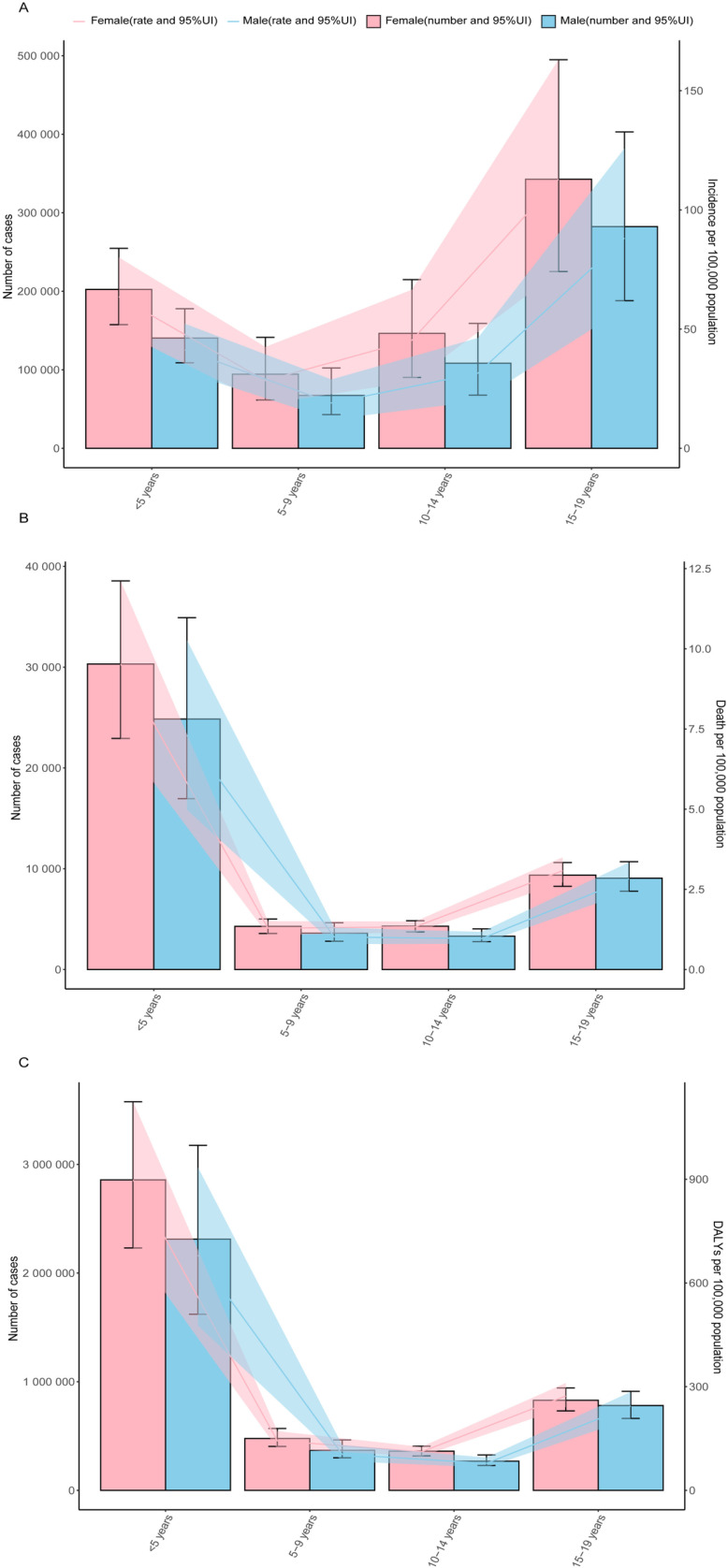
Incidence, death and DALYs of TB, by age and sex in 2021. **(A)** **Number of Incidence cases globally and Incidence per 100,000 population(B)** **Number of Death cases globally and Mortality per 100,000 population(C)** **Number of DALYs cases globally and DALYs per 100,000 population.** Lines indicate prevalent case with 95% uncertainty intervals for men and women.

In 2021, the global incidence, death and DALYs of TB among individuals under 20 years of age exhibited notable age and sex variations. The incidence rate was highest among individuals aged 15-19 years old, for both males (88.16, 95% UI:58.71-125.79) and females (112.87, 95% UI: 74.15-162.99). Among children under 5 years, there was a decrease in incidence rate in females(63.58, 95% UI: 49.49-80.05) and males(41.30, 95% UI: 32.03-52.28)(Fig 2A). In 2021, the global TB death rate reached its highest level in the youngest age group (<5 years)(Females:9.53, 95% UI: 7.21-12.12;Males: 7.31, 95% UI: 4.99-10.27) and was higher in females across all age groups. Following this, in males aged 5-9 and 10-14 years old, the mortality rate gradually decreases, then rises again with increasing age (Fig 2B). Similarly, the global TB DALYs rate was highest among individuals aged <5 years old(Females: 898.17, 95% UI: 701.18-1124.07; Males: 680.24, 95% UI: 476.81-934.06), and then decreased with advancing age, for both males and females. However, in adolescents aged 15-19 years old, DALYs rate begin to increase again (Fig 2C). Moreover, in all age groups, females have higher number of incidence, mortality, and DALYs compared to males.

### Regional trend

**Fig 3:**
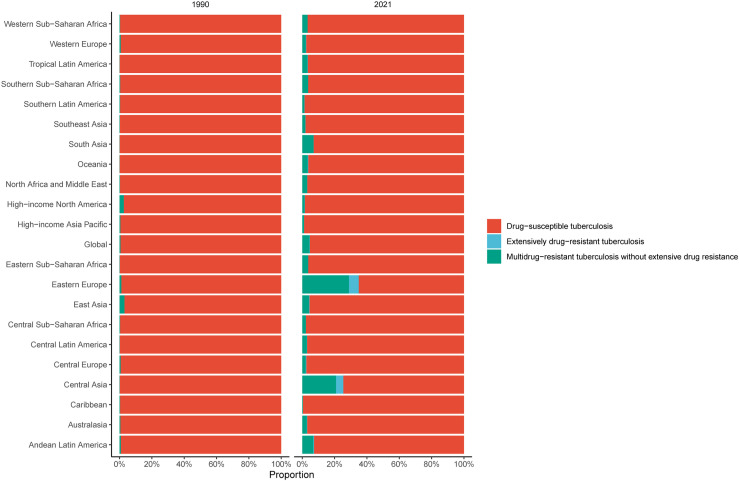
Composition ratio of each type of tuberculosis age-standardized incidence rate in 21 regions in 1990 and 2021.

In 2021, Southern Sub-Saharan Africa (302.88), Central Sub-Saharan Africa (164.95), and Eastern Sub-Saharan Africa (106.21) had the highest ASIR for TB (per 100,000), the proportion of MDR in 3 regions was 3.66%, 2.24%, 3.73% respectively, whereas High-income North America (1.05), Australasia (2.38), and High-income Asia Pacific (2.76) had the lowest (Table 1, Fig 3, S2 Table).Central Sub-Saharan Africa (14.07), Southern Sub-Saharan Africa (10.28), and Eastern Sub-Saharan Africa (8.62) had the highest ASMR from TB (per 100,000) in individuals under 20 years in 2021, and the proportion of MDR was 5.30%, 9.57%, 9.57% respectively (Table 2, S1 Fig, S3 Table).Similarly, Central Sub-Saharan Africa (1308.15), Southern Sub-Saharan Africa (1011.34), and Eastern Sub-Saharan Africa (800.29) had the highest ASDR from TB per 100,000 population, the proportion of MDR in 3 regions was 4.78%, 8.64%, 8.30% respectively (S1 Table, S2 Fig, S4 Table).

From 1990 to 2021, the largest decreases in the ASIR of TB were found in High-income Asia Pacific (-78.0%), Andean Latin America and East Asia (-75.5%), and North Africa and Middle East (-66.1%) (Table 1). At the same time, the ASDR of TB among individuals under 20 years showed a decrease in different regions. The greatest decreases in High-income Asia Pacific (−97.0%), East Asia (−97.7%), and Andean Latin America (−94.0%) (Table 2). The DALYs showed a significant decrease in all regions from 1990 to 2021, with the largest decreases in High-income Asia Pacific (−95.8%), East Asia (−95.4%), and Andean Latin America (−93.6%) (S1 Table).

### National trend

**Fig 4:**
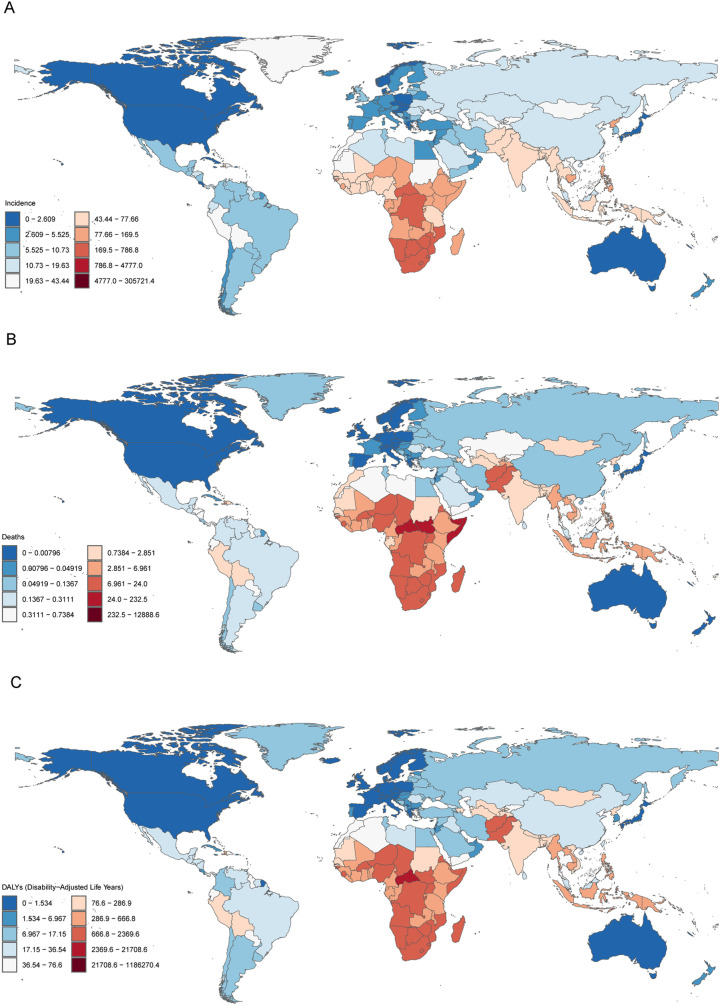
The incidence, death and DALYs in individuals aged 0 to 19 years in 204 countries and territories in 2021. **(A) incidence number (B) death number** **(C)** **DALYs number.** DALYs, disability-adjusted life years.

Among all the 204 countries and territories, India still reported a high incidence, although the number of cases had decreased to 305,721.35 (95% UI: 221,340.94–405,213.14). Similarly, Nigeria reported 87,340.14 cases (95% UI: 68,819.18–107,159.19) in 2021. Lesotho (409.07 per 100,000 population) had the highest ASIR of TB (Fig 4A and S5 Table). India and Nigeria also reported the highest number of death cases, 12,888.61 (95% UI: 10,616.29–16,004.70) and 12,331.75 (95% UI: 7,317.10–18,443.71) respectively. The national ASMR for TB in 2021 varied from 0.001 to 51.592 mortality per 100, 000 population. The highest rates were seen in Central African Republic (51.59), Somalia (26.34), and the South Sudan (25.85), whereas the lowest rates were found in Andorra (0.001), Sweden (0.001), and Denmark (0.002) (Fig 4B and S6 Table). In2021, the global burden of TB measured in DALYs for individuals under 20 years was dominated by India, which reported 1186,270.36 cases (95% UI: 987,064.08–1473,281.40). Nigeria followed with 1122,978.823 cases (95% UI: 681,848.58–1644,478.00). The highest ASDR were seen in Central African Republic (4,610.30), Somalia (2,349.80), and South Sudan (2,329.39) whereas the lowest rates were in Andorra (0.17), San Marino (0.60), and South Sudan (0.68) (Fig 4C and S7 Table).

### Association with the socio-demographic index

**Fig 5:**
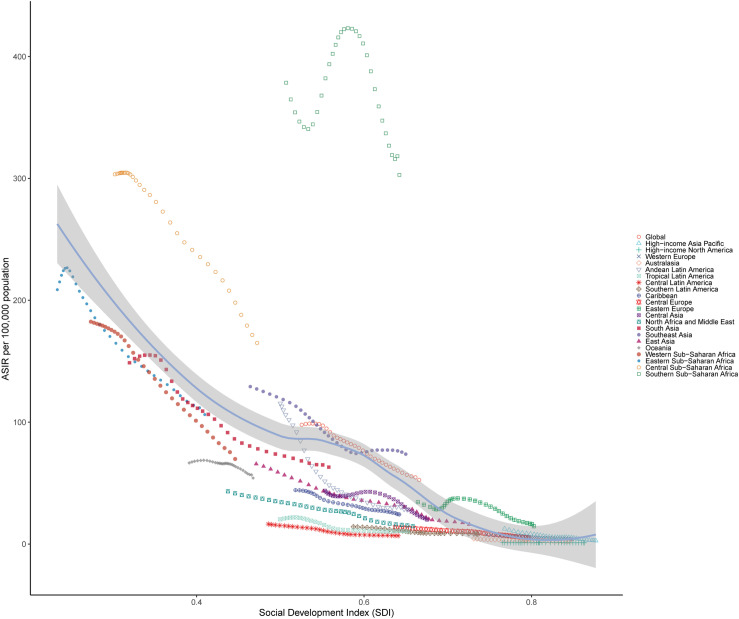
Age standardized incidence rates of Tuberculosis for the 21 Global Burden of Disease regions by SDI, 1990-2021. ASIR, age standardized incidence rates; SDI, socio-demographic index. Thirty-two points are plotted for each region and show the observed ASIR from 1990 to 2021 for that region. Expected values, based on SDI and disease rates in all locations, are shown as a solid line.

At the regional level, there was a negative association between SDI and the ASIR of TB, suggesting that the burden of TB was lower in regions with higher SDI development. However, Southern Sub−Saharan Africa was an exception, with much higher than predicted levels, and showing a significant increase. In contrast, Central Latin America, Tropical Latin America, North Africa and Middle East, Caribbean, Oceania and High-income North America had lower than expected burdens from 1990 to 2021(Fig 5). As for ASMR of tuberculosis, most regions experienced reductions or remained stable, except for Southern Sub-Saharan Africa, where rates first increased and then decreased. In general, the ASMR of TB and SDI levels are inversely correlated, with regions of higher SDI typically exhibiting lower ASMR (S3 Fig). From 1990 to 2021, the ASDR decreased exponentially with increases in SDI. In low-SDI regions, such as Central Sub−Saharan Africa and Eastern Sub−Saharan Africa had higher ASDR. This starkly contrasts with high-income regions, where ASDR were substantially lower (S4 Fig). Furthermore, distinct patterns were observed in many medium SDI regions. From 1990 to 2021, some localities kept TB ASDR substantially below projected values, while others significantly exceeded them.

## Discussion

To our knowledge, this is the first study to estimate the TB burden among children and adolescents under the age of 20. Using data from the GBD 2021, we present the latest information on TB prevalence, mortality, and DALYs for the 0-19 age group from 1990 to 2021, as well as ASR for 204 countries and territories. In 2021, TB in children and adolescents was responsible for 1.38 million incidence cases, 89,100 deaths, and 8.26 million DALYs. We observed an overall decline in ASIR, ASMR, and ASDR from 1990 to 2021. However, in 2021, the incidence, mortality, and DALYs of MDR-TB and XDR-TB among people aged 0-19 increased compared to 1990.

A recent study reported that between 1990 and 2019, the incidence, prevalence, and DALYs of TB among individuals aged 10-24 years decreased by 1.28%, 3.06%, and 2.83%, respectively [10]. Our analysis discovered that from 1990 to 2021, the global incidence, death, and DALYs of TB among people under the age of 20 dropped by 2.16%, 3.90%, and 4.21%, respectively. These findings emphasize the progress made over the last three decades in lowering TB among children and adolescents worldwide. However, the data also highlight the importance of continuing efforts to address the residual burden, particularly in improving TB diagnosis, treatment, and preventive.

According to the third edition of the Roadmap towards ending TB in children and adolescents, about 1.25 million children and adolescents (0-14 years old) globally had TB in 2022, but more than half (51%) of these cases were not diagnosed or reported [22]. The WHO report on the United Nations High-Level Meeting treatment targets for TB, by the end of 2021, only 54% of the 3.5 million children who require TB treatment would receive it by the end of 2021[3]. Among children with rifampicin-resistant or multidrug-resistant tuberculosis, the treatment coverage was just 15%. Consequently, the burden of TB in children and adolescents may be underestimated, and current prevention and treatment efforts are unlikely to meet expectations.

Notably, several studies have found sex differences in the burden of TB. One study indicated that during adolescence, the proportion of females developing both pulmonary and extrapulmonary TB is higher than that of males [23]. For instance, data from Gauteng Province in South Africa suggest that females aged 10 to 19 have a higher proportion of TB cases than males [24]. Similarly, our study reveals that from 1990 to 2021, the ASIR, ASMR, and ASDR of TB were higher among females under the age of 20 than males of the same age group. However, Zhang et al. reported that in the general population, the ASDR of TB is lower in females than in males [20]. Another global epidemiological study also found that males have higher incidence and mortality rates of TB than females [25]. In general, females are more likely than males to develop from TB infection to active disease throughout adolescence, even though more males are diagnosed with the disease each year globally.

There could be a number of reasons for the increased incidence of TB in teenage girls. Firstly, immunological aspects are important. Males and females have significant immunological differences during adolescence, which could be related to different exposure levels to sex hormones. In women, inflammation is often stronger due to the immune-stimulating effects of estrogen in particular. Additionally, females are more susceptible to TB due to a higher Th2 imbalanced response [5,26]. Secondly, non-immunological elements including sexual behavior and concurrent infections also play a role. Pregnancy can result from sexual activity, which lowers cell-mediated immunity and raises the occurrence of TB [27,28]. Globally, HIV infection is a substantial risk factor for TB in females. In the 10 countries where HIV infection rates account for two-thirds of the global total, 59% of those infected are females, with adolescent girls and young women aged 15-24 being particularly vulnerable [29]. Consequently, teenage girls have a higher risk of developing TB than their male peers.

According to our study, there was a notable increase in the mortality rate and DALYs due to MDR-TB and XDR-TB among children and adolescents in 2021 compared to 2019. The three regions with the greatest increases were Eastern Europe, Central Asia, and Andean Latin America. This may be attributed to the inadequacies in these regions’ healthcare systems, limited TB diagnostic and treatment capacities, and socioeconomic factors such as poverty, malnutrition, and high population density, which all exacerbate the spread of drug-resistant TB [30,31]. A 2021 systematic review that compiled 37 studies over the past two decades estimated that the global proportion of drug-resistant TB in children is as high as 13.59%. Notably, the prevalence of MDR-TB in high-income countries (1.8%) was significantly lower than in lower-middle-income (6.3%) and upper-middle-income countries (7.3%) [32]. MDR-TB poses a severe threat to the health of children and adolescents, with high treatment costs and low success rates, making it difficult to manage drug-resistant TB in these populations. As a result, in regions with a high burden of pediatric MDR-TB, it is imperative to promote emerging drug resistance detection methods [33], implement preventive treatment strategies [34], and enhance psychosocial interventions [35] to alleviate disease burden.

Existing research has found a bimodal pattern in the risk of TB among children, with children under five years old and adolescents having considerably elevated risks of disease progression and mortality after initial infection [36]. Our study suggested that in 2021, the mortality rate and DALYs for TB peaked among children under the age of five, followed by adolescents aged 15 to 19. Conversely, adolescents aged 15 to 19 had a higher incidence of TB than children under the age of five. This finding supports previous reports, further confirming the bimodal pattern of TB burden in children and adolescents.

This bimodal pattern may be attributed to difficulties in diagnosing and treating TB in children. When compared to adults, children with TB frequently lack typical clinical signs such as cough and hemoptysis. The diverse and nonspecific clinical presentations make TB diagnosis in children significantly more challenging [37]. Furthermore, the difficulty in collecting specimens in children contributes significantly to the difficulties in acquiring microbiological evidence of TB. Young children often have poor sputum production, low fluid volumes, and difficulty in collecting samples like gastric lavage and bronchoalveolar lavage fluids, compounded by poor cooperation, all of which increase the difficulty of obtaining microbiological diagnostic evidence [38]. Pediatric TB patients have a low bacterial load, which results in a lower positive rate on microbiological tests compared to adults. Although the X-pert MTB/RIF assay has shown higher positivity rates in diagnosing pediatric TB than traditional smear and culture methods, its sensitivity remains low, ranging from 51% to 66% [39,40], indicating that additional development is required. In terms of treatment, there is currently no pediatric-specific anti-tuberculosis medication formulations available. Clinical dosing is frequently based on scaled-down adult formulations, which might result in erroneous dosing and reduce therapy efficacy [41]. Furthermore, the lack of pharmacokinetic and pharmacodynamic data for children also influences therapy outcomes. Insufficient medication concentrations can limit therapeutic efficacy while also encouraging the development of drug resistance. Therefore, the development of new diagnostic technologies and treatment medications is crucial to alleviating the TB burden in children.

Our findings show a significant negative correlation between TB-related ASIR, ASMR, ASDR and SDI. This suggests that regions with higher socioeconomic levels tend to have a lower TB burden. Similarly, the WHO reported that TB infections in 2022 were predominantly concentrated in low-income and middle-income countries, including Southeast Asia (46%), Africa (23%), and the Western Pacific (18%) [42]. This regional distribution pattern highlights the socioeconomic inequalities of TB on a global scale. According to the GBD 2021, excluding the three primary TB risk factors of smoking, alcohol consumption, and diabetes, the number of TB deaths in Central Europe, Eastern Europe, and Central Asia is expected to decrease by 66.3% from 2015 to 2020[43]. Obore N et al. explicitly pointed out that smoking doubles the risk of TB (RR = 2.67, 95% CI 2.017-3.527), while exposure to second-hand tobacco smoke raises the risk of TB infection by twofold (RR = 2.15, 95% CI 1.419-3.242) [44]. Another study revealed that alcohol has a significant impact on TB incidence and death, notably in Africa and Southeast Asia [45]. The widespread exposure to these risk factors in countries with low and middle SDI highlights the need for targeted interventions in these regions, such as enhanced disease awareness campaigns and screening of high-risk populations, to interrupt the transmission of TB at its source and reduce the TB burden [46].

In addition to these individual risk factors, SDI serves as a composite assessment of a country’s poverty status. Countries with lower SDI generally have difficulty accessing healthcare services, particularly the availability and affordability of TB diagnosis and treatment [47]. The interplay between socioeconomic development and TB burden underscores the necessity of formulating and implementing strategic interventions tailored to the specific challenges of different regions.

The COVID-19 pandemic has had a profound impact on the accessibility of TB diagnostic and treatment services and the TB burden worldwide. The uneven allocation of global health resources disrupted TB diagnosis, treatment, and prevention efforts. According to the Global Tuberculosis Report 2023, newly diagnosed TB cases dropped sharply from 7.1 million in 2019 to 5.8 million in 2020, and then to 6.4 million in 2021, only returning to 7.5 million in 2022—the highest figure since the WHO began global TB monitoring in 1995[42]. During the pandemic, extensive healthcare resources were diverted to address COVID-19, delaying TB screening efforts in many countries. This delay may have been particularly severe in children and adolescents, as school closures and social distancing measures potentially altered TB transmission patterns in these groups. GBD 2021 data indicates that TB incidence and mortality declined in 2020 and 2021 compared to 2019, likely reflecting not an actual decrease in TB incidence but rather underreporting and delayed diagnosis. Additionally, co-infection with COVID-19 may have raised mortality and complication rates among TB patients. While this study does not delve into specific data on COVID-19 and TB co-infections, existing research suggests that co-infection with COVID-19 can exacerbate patient outcomes [48]. Therefore, despite the reported decrease in TB cases and mortality during the pandemic, the true burden of TB might have been underestimated due to diagnostic and reporting delays. This conclusion emphasizes the importance of maintaining continuity in TB control efforts during future pandemics.

## Strengths and limitations

Overall, the article’s strengths include its broad geographic coverage, emphasis on a vulnerable population, in-depth analysis of drug-resistant tuberculosis, and inclusion of socioeconomic factors. These provide a strong foundation for guiding future research and policy. Despite the strong findings presented in this study, it is important to acknowledge several limitations that may affect the interpretation of the results. First, this study is based on data from GBD 2021, and the accuracy of the estimates is determined on the quality of the underlying data. The study’s capacity to comprehensively reflect the global burden of TB in children and adolescents may be limited due to insufficient data for some regions or groups. Second, there are diagnostic and reporting factors: diagnosing TB in children presents significant challenges, including the risks of underdiagnosis and misdiagnosis. In addition, differences in diagnostic criteria, reporting standards, and data collection methods between countries and regions may impact the accuracy of the results. Third, temporal factors must be considered. This analysis spans over a 30-year period, during which substantial developments in medical technologies, treatment protocols, and public health policies may have influenced on the burden of TB. Fourth, socioeconomic factors such as household income, education level, and access to healthcare facilities may affect the incidence and treatment outcomes of TB in children and adolescents. Finally, intervention-related factors should be noted. Over the past several decades, global and national governments have implemented different public health interventions to combat TB, and the effectiveness of these interventions may vary by region. This study may not have adequately assessed the impact of these interventions on the burden of TB.

## Future directions

Recommendations for future research and policy are crucial to maintaining and enhancing TB control. Further research should focus on understanding the underlying causes contributing to anomalies in regions such as sub-Saharan Africa and exploring effective interventions tailored to local conditions. This may include investigating the sociocultural, economic, and environmental factors influencing TB transmission and progression. Policy initiatives should prioritize strengthening healthcare infrastructure and accessibility in low and middle SDI regions, ensuring universal access to diagnostic and treatment services. Implementing socioeconomic development plans to improve living conditions, nutrition, and education levels, as well as focusing on finding new vaccines, treatment drugs, and diagnostic methods, will play a pivotal role in reducing TB burden among children and adolescents.

## Conclusion

In conclusion, while the decrease in TB burden among those under 20 years old is encouraging, it reflects the success of global TB control strategies, including enhanced diagnostic capabilities, effective treatment regimens, widespread vaccination efforts, and broader socioeconomic improvements. However, TB remains in certain regions and populations, demanding continued and strengthened efforts to address remaining challenges. By focusing on regional and demographic disparities, continuing to invest in healthcare infrastructure, improving TB diagnosis, prevention, and treatment technologies, we can move closer to the goal of eliminating TB and ultimately ensure a healthier future for all young people worldwide.

## Supporting information

S1 Fig Composition ratio of each types of tuberculosis ASMR in 1990 and 2021.
(TIF)

S2 Fig Composition ratio of each types of tuberculosis ASDR in 1990 and 2021.
(TIF)

S3 Fig ASMR of Tuberculosis for the 21 Global Burden of Disease regions by SDI, 1990-2021.
Thirty-two points are plotted for each region and show the observed ASMR from 1990 to 2021 for that region. Expected values, based on socio-demographic index and disease rates in all locations, are shown as a solid line.
(TIF)

S4 Fig ASDR of Tuberculosis for the 21 Global Burden of Disease regions by SDI, 1990-2021.
Thirty-two points are plotted for each region and show the observed ASDR from 1990 to 2021 for that region. Expected values, based on socio-demographic index and disease rates in all locations, are shown as a solid line.
(TIF)

S1 Table DALYs, ASDR, and Relative change of Tuberculosis in children and Adolescence in global and 21 regions, with EAPC from 1990 and 2021.
(XLSX)

S2 Table Each types of tuberculosis ASIR in 1990 and 2021.
(XLSX)

S3 Table Each types of tuberculosis ASMR in 1990 and 2021.
(XLSX)

S4 Table Each types of tuberculosis ASDR in 1990 and 2021.
(XLSX)

S5 Table Incidence number, ASIR under the age of 20 in 204 countries and territories in 2021.
(XLSX)

S6 Table Death number, ASMR under the age of 20 in 204 countries and territories in 2021.
(XLSX)

S7 Table DALYs number, ASDR under the age of 20 in 204 countries and territories in 2021.
(XLSX)

S1 Appendix The essential code used for the analysis.
(DOCX)
