# Supplementary figures and images for: Tuberculosis incidence, deaths and disability-adjusted life years in children and adolescence, 1990–2021: Results from the Global Burden of Disease Study 2021
Source: PLoS One. 2025 Mar 10;20(3):e0317880. doi: 10.1371/journal.pone.0317880 (PMC11892809; doi:10.1371/journal.pone.0317880)

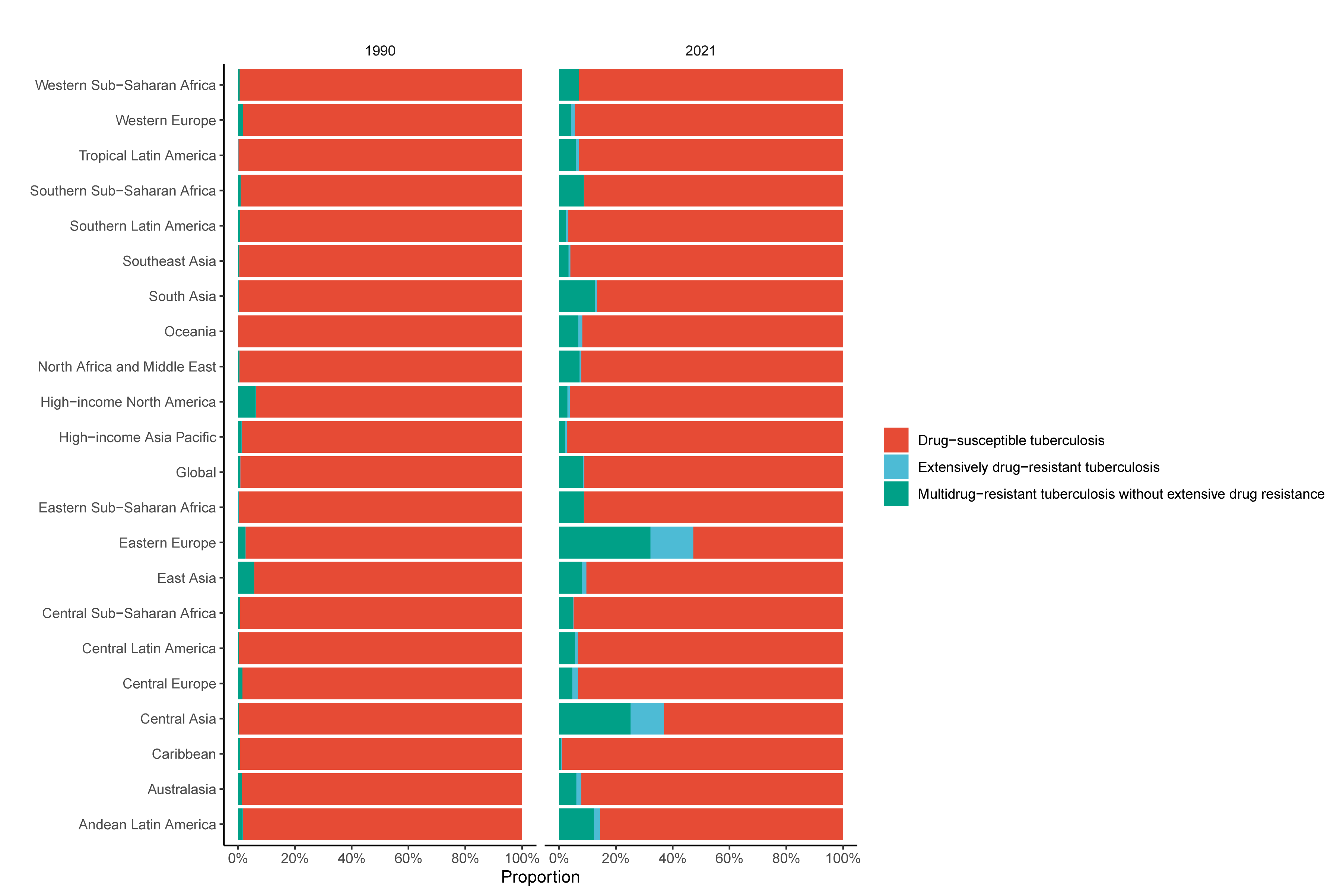

Supplement: S1 Fig — (TIF) [file pone.0317880.s001.tif]

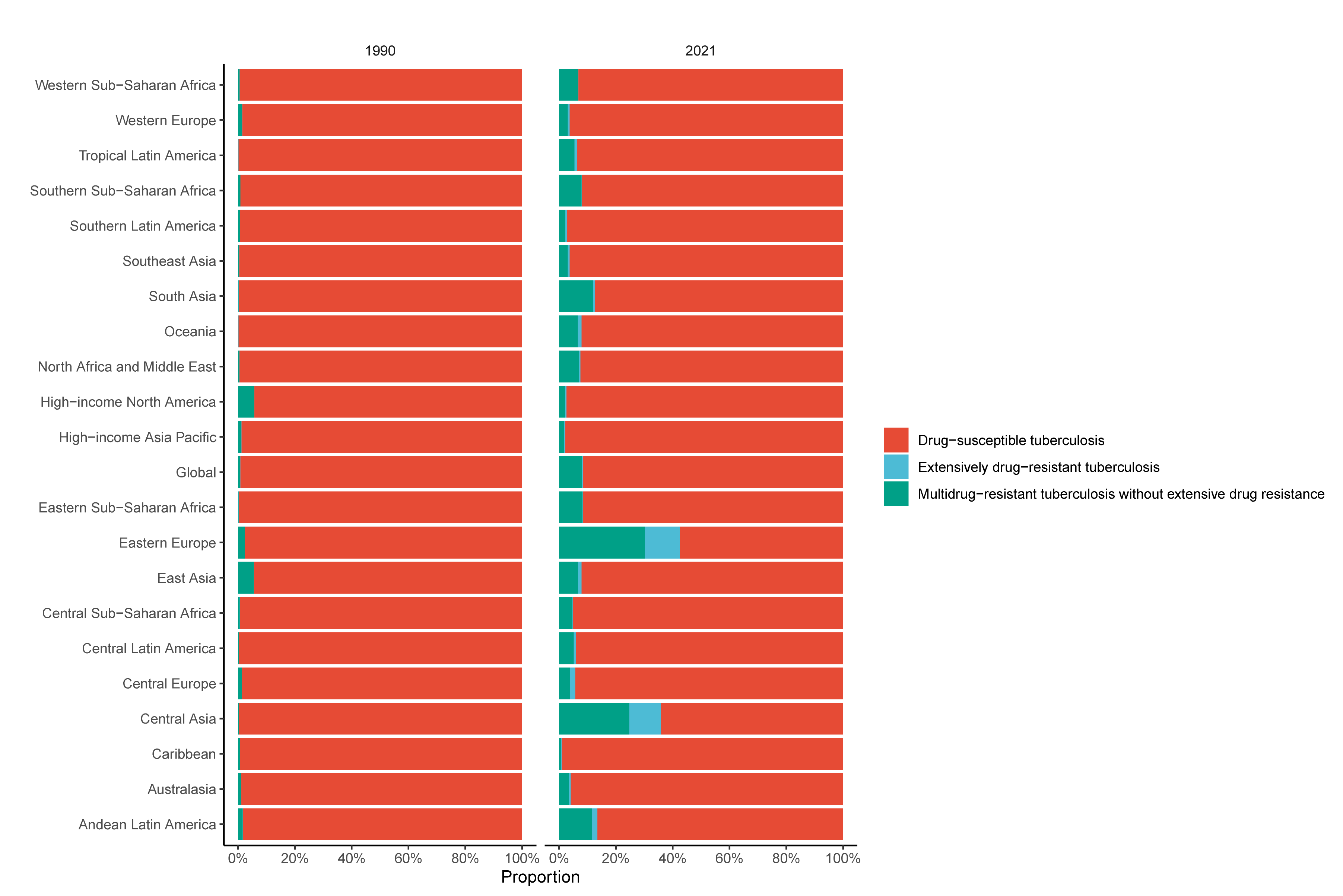

Supplement: S2 Fig — (TIF) [file pone.0317880.s002.tif]

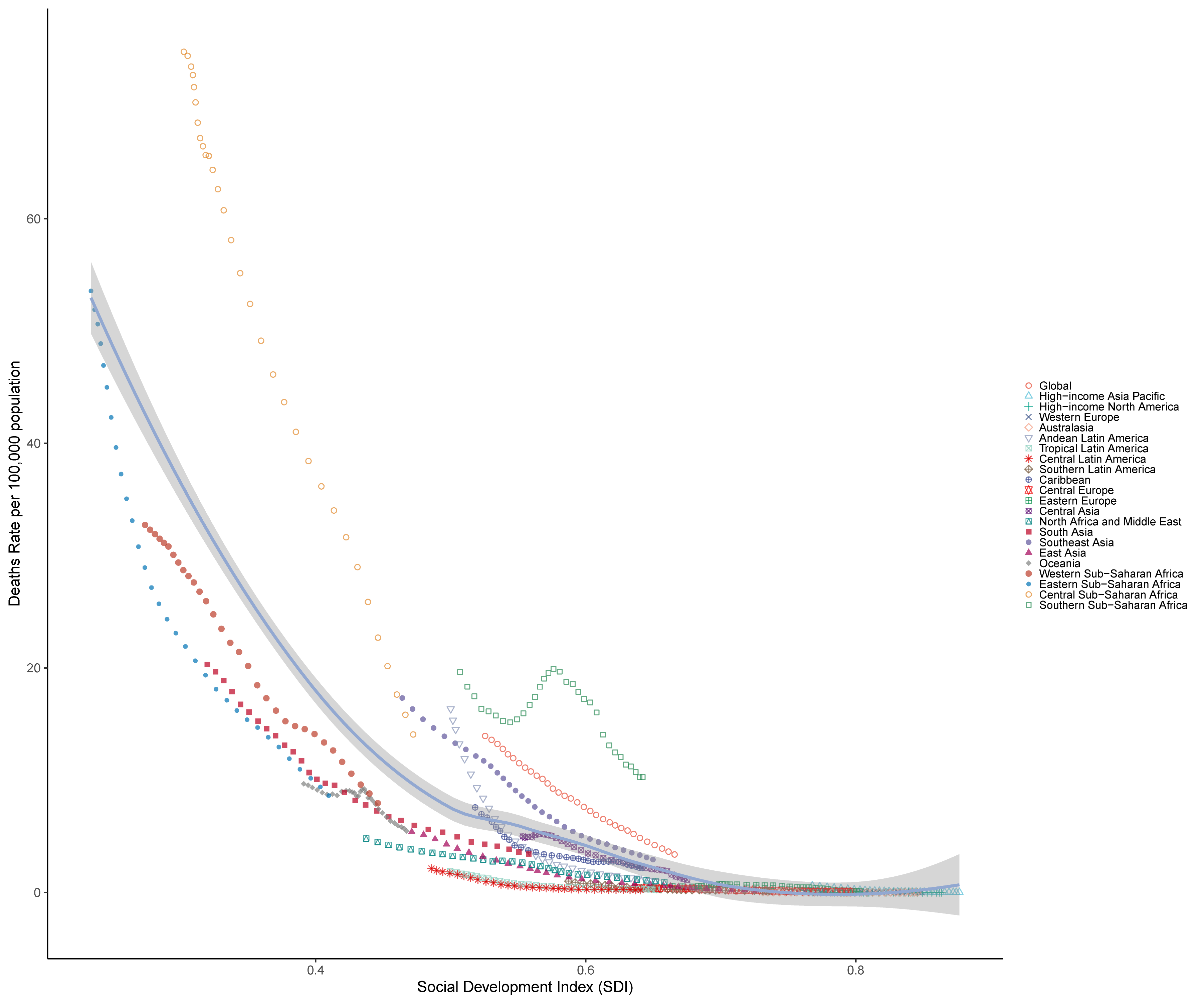

Supplement: S3 Fig — Thirty-two points are plotted for each region and show the observed ASMR from 1990 to 2021 for that region. Expected values, based on socio-demographic index and disease rates in all locations, are shown as a solid line. (TIF) [file pone.0317880.s003.tif]

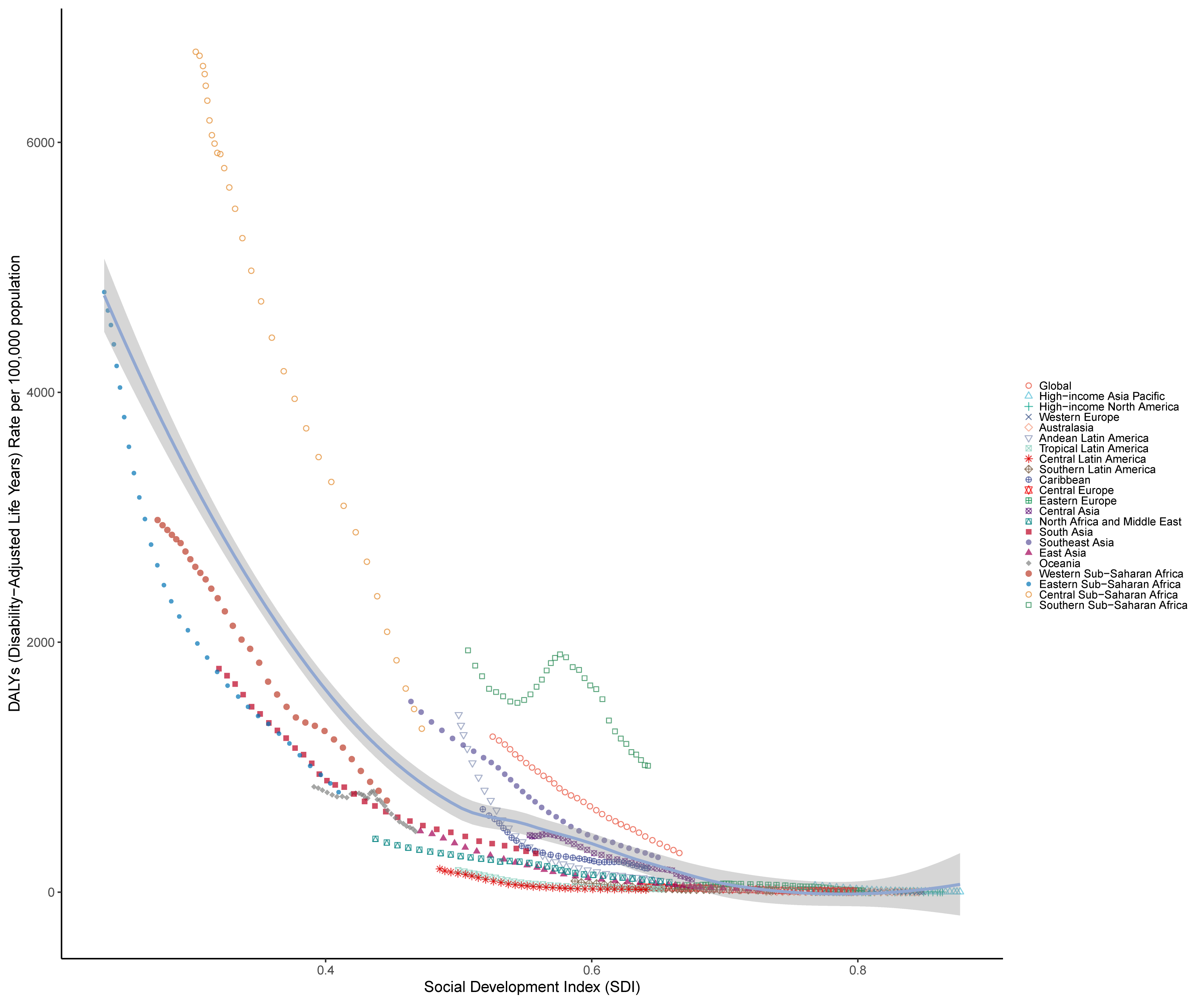

Supplement: S4 Fig — Thirty-two points are plotted for each region and show the observed ASDR from 1990 to 2021 for that region. Expected values, based on socio-demographic index and disease rates in all locations, are shown as a solid line. (TIF) [file pone.0317880.s004.tif]
