# Supplementary material for: Tuberculosis incidence, deaths and disability-adjusted life years in children and adolescence, 1990–2021: Results from the Global Burden of Disease Study 2021
Source: PLoS One. 2025 Mar 10;20(3):e0317880. doi: 10.1371/journal.pone.0317880 (PMC11892809; doi:10.1371/journal.pone.0317880)
Supplement: S1 Appendix — (DOCX) [file pone.0317880.s012.docx]

**Appendix of Code**

library(dplyr)

###21+1+5SDI data merge

df1 <- read.csv("1.csv")

df2 <- read.csv("2.csv")

df3 <- read.csv("3.csv")

dfall <- bind_rows(df1, df2, df3)

write.csv(dfall,"21+1+5SDI.csv",row.names = FALSE)

library(scales)

library(sf)

library(tidyverse)

library(ggrepel)

library(ggsci)

library(patchwork)

library(data.table)

load("/Users/mac/Desktop/GBD最新版本/shinyGBD_V2.27/GBD.Rdata")

load("/Users/mac/Desktop/GBD最新版本/shinyGBD_V2.27/GBDpop1990_2100.Rdata")

load("/Users/mac/Desktop/GBD最新版本/shinyGBD_V2.27/population231.Rdata")

df <- read.csv("21+1+5SDI.csv")

head(df)

for (i in unique(df$measure_name)) {

dfx <- df %>%

filter(measure_name==i) %>%

filter(cause_name=="Tuberculosis") %>%

filter(location_name=="Global") %>%

filter(age_name=="<20 years") %>%

filter(year %in%c(1990,2021)) %>%

filter(!metric_name=="Percent") %>%

mutate(x = paste0(year,"-", sex_name,"-",metric_name)) %>%

mutate(num = if_else(metric_name == "Number",

sprintf("%.2f (%.2f, %.2f)", val, lower, upper),

sprintf("%.2f (%.2f, %.2f)", val, lower, upper))) %>%

select(2,4,6,8,10,12,14:18)

write.csv(dfx,paste0("global-sex-",i,".csv"))

}

for (i in unique(df$measure_name)) {

dfx <- df %>%

filter(measure_name==i) %>%

filter(cause_name=="Tuberculosis") %>%

filter(location_name=="Global") %>%

filter(sex_name=="Both") %>%

filter(year %in%c(1990,2021)) %>%

filter(!metric_name=="Percent") %>%

mutate(x = paste0(year,"-", age_name,"-",metric_name)) %>%

mutate(num = if_else(metric_name == "Number",

sprintf("%.2f (%.2f, %.2f)", val, lower, upper),

sprintf("%.2f (%.2f, %.2f)", val, lower, upper))) %>%

select(2,4,6,8,10,12,14:18)

write.csv(dfx,paste0("global-age_name-",i,".csv"))

}

for (i in unique(df$measure_name)) {

dfx <- df %>%

filter(measure_name==i) %>%

filter(cause_name=="Tuberculosis") %>%

filter(location_id %in% name_5) %>%

filter(sex_name=="Both") %>%

filter(age_name=="<20 years") %>%

filter(year %in%c(1990,2021)) %>%

filter(!metric_name=="Percent") %>%

mutate(x = paste0(year,"-", location_name,"-",metric_name)) %>%

mutate(num = if_else(metric_name == "Number",

sprintf("%.2f (%.2f, %.2f)", val, lower, upper),

sprintf("%.2f (%.2f, %.2f)", val, lower, upper))) %>%

select(2,4,6,8,10,12,14:18)

write.csv(dfx,paste0("global-5SDI-",i,".csv"))

}

library(dplyr)

# Create an empty data frame to store all results

final_results <- data.frame()

# Iterate over different measure_name and sex_name

for (i in unique(df$measure_name)) {

for (j in unique(df$sex_name)) {

# Filter and clean the data

df2 <- df %>%

filter(measure_name == i) %>%

filter(sex_name == j) %>%

filter(location_name == "Global") %>%

filter(age_name == "<20 years") %>%

filter(metric_name != "Percent") %>%

filter(!is.na(val), !is.infinite(val), val > 0)

if (nrow(df2) > 1) {

lm_fit <- lm(log(val) ~ year, data = df2)

beta <- coef(lm_fit)["year"]

se_beta <- summary(lm_fit)$coefficients["year", "Std. Error"]

EAPC <- (exp(beta) - 1) * 100

lower_CI <- (exp(beta - 1.96 * se_beta) - 1) * 100

upper_CI <- (exp(beta + 1.96 * se_beta) - 1) * 100

eapc_formatted <- sprintf("%.2f (%.2f, %.2f)", EAPC, lower_CI, upper_CI)

final_results <- rbind(final_results, data.frame(

Measure = i,

Sex = j,

EAPC = eapc_formatted

))

}

}

}

# Save results as CSV file

write.csv(final_results, file = "EAPC-sex_results.csv", row.names = FALSE)

# Output results to console

print(final_results)

library(dplyr)

# Create an empty data frame to store all results

final_results <- data.frame()

# Iterate over different measure_name and age_name

for (i in unique(df$measure_name)) {

for (j in unique(df$age_name)) {

# Filter and clean the data

df2 <- df %>%

filter(measure_name == i) %>%

filter(age_name == j) %>%

filter(location_name == "Global") %>%

filter(sex_name == "Both") %>%

filter(metric_name != "Percent") %>%

filter(!is.na(val), !is.infinite(val), val > 0)

if (nrow(df2) > 1) {

lm_fit <- lm(log(val) ~ year, data = df2)

beta <- coef(lm_fit)["year"]

se_beta <- summary(lm_fit)$coefficients["year", "Std. Error"]

EAPC <- (exp(beta) - 1) * 100

lower_CI <- (exp(beta - 1.96 * se_beta) - 1) * 100

upper_CI <- (exp(beta + 1.96 * se_beta) - 1) * 100

eapc_formatted <- sprintf("%.2f (%.2f, %.2f)", EAPC, lower_CI, upper_CI)

final_results <- rbind(final_results, data.frame(

Measure = i,

Agegroup = j,

EAPC = eapc_formatted

))

}

}

}

# Save results as CSV file

write.csv(final_results, file = "EAPC-agegroup_results.csv", row.names = FALSE)

# Output results to console

print(final_results)

library(dplyr)

# Create an empty data frame to store all results

final_results <- data.frame()

# Iterate over different measure_name and location_name

for (i in unique(df$measure_name)) {

for (j in unique(df$location_name)) {

# Filter and clean the data

df2 <- df %>%

filter(location_id %in% name_5) %>%

filter(measure_name == i) %>%

filter(location_name == j) %>%

filter(age_name == "<20 years") %>%

filter(sex_name == "Both") %>%

filter(metric_name != "Percent") %>%

filter(!is.na(val), !is.infinite(val), val > 0)

if (nrow(df2) > 1) {

lm_fit <- lm(log(val) ~ year, data = df2)

beta <- coef(lm_fit)["year"]

se_beta <- summary(lm_fit)$coefficients["year", "Std. Error"]

EAPC <- (exp(beta) - 1) * 100

lower_CI <- (exp(beta - 1.96 * se_beta) - 1) * 100

upper_CI <- (exp(beta + 1.96 * se_beta) - 1) * 100

eapc_formatted <- sprintf("%.2f (%.2f, %.2f)", EAPC, lower_CI, upper_CI)

final_results <- rbind(final_results, data.frame(

Measure = i,

SDI = j,

EAPC = eapc_formatted

))

}

}

}

# Save results as CSV file

write.csv(final_results, file = "EAPC-SDI_results.csv", row.names = FALSE)

# Output results to console

print(final_results)

df <- read.csv("pc.csv")

head(df)

dfx <- df %>%

filter(cause_name=="Tuberculosis") %>%

filter(location_name=="Global") %>%

filter(age_name=="<20 years") %>%

filter(!metric_name=="Percent") %>%

mutate(PC = sprintf("%.2f%%", val*100)) %>%

select(2,4,6,8,10,12,14:18)

write.csv(dfx,paste0("PC-sex.csv"))

dfx <- df %>%

filter(cause_name=="Tuberculosis") %>%

filter(location_name=="Global") %>%

filter(sex_name=="Both") %>%

filter(!metric_name=="Percent") %>%

mutate(PC = sprintf("%.2f%%", val*100)) %>%

select(2,4,6,8,10,12,14,18)

write.csv(dfx,paste0("PC-agegroup.csv"))

dfx <- df %>%

filter(cause_name=="Tuberculosis") %>%

filter(sex_name=="Both") %>%

filter(location_id %in% name_5) %>%

filter(age_name=="<20 years") %>%

filter(!metric_name=="Percent") %>%

mutate(PC = sprintf("%.2f%%", val*100)) %>%

select(2,4,6,8,10,12,14,18)

write.csv(dfx,paste0("PC-5SDI.csv"))

######################################################################Fig1

df <- read.csv("21+1+5SDI.csv")

head(df)

dfx <- df %>%

filter(measure_name == "Incidence") %>%

filter(cause_name == "Tuberculosis") %>%

filter(location_name == "Global") %>%

filter(age_name == "<20 years") %>%

filter(metric_name == "Rate")

byx <- 5

p <- ggplot(dfx, aes(x = year, y = val, group = sex_name)) +

geom_ribbon(aes(ymin = lower, ymax = upper, fill = sex_name), alpha = 0.2, show.legend = TRUE) +

geom_line(aes(color = sex_name, linetype = sex_name)) +

theme_classic() +

scale_y_continuous(labels = label_number(unit = "K")) +

scale_x_continuous(

breaks = seq(min(dfx$year, na.rm = TRUE), max(dfx$year, na.rm = TRUE), by = byx)

) +

labs(x = "Year", y = "ASIR per 100,000 population", color = "Sex", linetype = "Sex") +

scale_color_lancet() +

scale_fill_lancet() +

guides(color = guide_legend(title = "Sex"), linetype = guide_legend(title = "Sex"), fill = guide_legend(title = "Sex")) +

theme(strip.background = element_blank())

# Print the chart

print(p)

write.csv(dfx,paste0("Fig1-2021-ASIR.csv"),row.names = FALSE)

ggsave(paste0("Fig1-2021-ASIR.pdf"),plot = p,width = 8,height = 6)

#####################################################################################Fig2

age_twofig=function(dfx=dfx,labx="Number of cases",color_scheme = 2){

color_palettes <- data.frame(

palette = c(1, 2, 3, 4, 5),

color1 = c("#E25A90", "lightpink", "#FF7E67", "#FFC0CB", "#EC7063"),

color2 = c("#58C1B2", "skyblue", "#15B8A0", "#D8BFD8", "#2471A3"),

legend_fill2 = rep("Male(number and 95%UI)",5),

legend_fill1 = rep("Female(number and 95%UI)",5),

legend_color2 = rep("Male(rate and 95%UI)",5),

legend_color1 = rep("Female(rate and 95%UI)",5)

)

dfx=dfx %>% mutate(age_name = if_else(age_name %in% c("80-84", "85-89", "90-94"),

paste(age_name, "years"),

age_name))

dfage=dfage %>% mutate(age_name = if_else(age_name %in% c("80-84", "85-89", "90-94"),

paste(age_name, "years"),

age_name))

library(ggplot2)

library(dplyr)

if (length(unique(dfx$age_id)) < 2 || length(unique(dfx$year)) > 1) {

p= ggplot() +

geom_text(aes(x = 0.5, y = 0.5, label = "Oops, your data is not suitable for this plot"), size = 12) +

theme_void()

return(p)

}

dfid=dfx %>% select(age_id,age_name) %>% distinct(age_id,.keep_all = T) %>%

left_join(.,dfage) %>% arrange(id)

dfx=left_join(dfx,dfid) %>% filter(!is.na(id))

dfx$age_name <- factor(dfx$age_name, levels = unique(dfid$age_name[order(dfid$id)]))

df1=dfx

df1_number <- df1 %>% filter(metric_name == "Number")

df1_rate <- df1 %>% filter(metric_name == "Rate")

max_number <- max(df1_number$val)

max_rate <- max(df1_rate$val)

scaling_factor <- max_number / max_rate

x1=color_palettes %>% filter(palette==color_scheme)

sexx=unique(dfx$sex_name)

if ("Male" %in% sexx && "Female" %in% sexx) {

x1=x1

} else if (sexx == "Male") {

x1$legend_fill2 <- "Male(number and 95%UI)"

x1$legend_fill1 <- "Male(number and 95%UI)"

x1$legend_color2 <- "Male(rate and 95%UI)"

x1$legend_color1 <- "Male(number and 95%UI)"

} else {

x1$legend_fill2 <- "Female(number and 95%UI)"

x1$legend_fill1 <- "Female(number and 95%UI)"

x1$legend_color2 <- "Female(rate and 95%UI)"

x1$legend_color1 <- "Female(number and 95%UI)"

}

p=ggplot() +

geom_bar(data = df1_number, aes(x = age_name, y = val, fill = sex_name),

stat = "identity", position = position_dodge(), color = "black") +

geom_errorbar(data = df1_number, aes(x = age_name, ymin = lower, ymax = upper, group = sex_name),

position = position_dodge(0.9), width = 0.25) +

geom_line(data = df1_rate, aes(x = age_name, y = val * scaling_factor, group = sex_name, color = sex_name),

position = position_dodge(0.9)) +

geom_ribbon(data = df1_rate, aes(x = age_name, ymin = lower * scaling_factor, ymax = upper * scaling_factor, group = sex_name, fill = sex_name),

alpha = 0.5, position = position_dodge(width = 0.9)) +

labs(x="",y = labx) +

scale_y_continuous(labels = label_number(unit = "K"), sec.axis = sec_axis(~./scaling_factor, name = "Incidence Rate per 100,000 population")) +

scale_fill_manual(values = c(x1$color1,x1$color2),labels =c(x1$legend_fill1,x1$legend_fill2),name=" ") +

scale_color_manual(values = c(x1$color1,x1$color2),labels = c(x1$legend_color1,x1$legend_color2),name=" ") +

theme_classic() +

theme(legend.position = "top",

axis.text.x = element_text(angle = 65, hjust = 1, size = 12, face = "bold"),

axis.text.y = element_text(size = 12, face = "bold"),

axis.title.x = element_text(size = 14, face = "bold"),

axis.title.y = element_text(size = 14, face = "bold"),

legend.text = element_text(size = 12, face = "bold"),

legend.title = element_text(size = 14, face = "bold")

)

return(p)

}

df <- read.csv("21+1+5SDI.csv")

head(df)

dfx <- df %>%

filter(measure_name == "Incidence") %>%

filter(cause_name == "Tuberculosis") %>%

filter(location_name == "Global") %>%

filter(sex_name != "Both") %>%

filter(age_id %in% c(1, 5:20, 30, 31, 32, 235)) %>%

filter(metric_name != "Percent") %>%

filter(year == 2021) %>%

mutate(age_name = factor(age_name, levels = c("<5 years", "5-9 years", "10-14 years", "15-19 years", "20-24 years",

"25-29 years", "30-34 years", "35-39 years", "40-44 years", "45-49 years",

"50-54 years", "55-59 years", "60-64 years", "65-69 years", "70-74 years",

"75-79 years", "80-84 years", "85-89 years", "90-94 years", "95+ years")))

xa = age_twofig(dfx = dfx, labx = paste0("Number of cases"), color_scheme = 2)

print(xa)

ggsave(paste0("Fig2-2021-agegroup-Incidence.pdf"), width = 12, height = 8)

write.csv(dfx, paste0("Fig2-2021-agegroup-Incidence.csv"))

##########################################################################Fig3

df <- read.csv("21+1+5SDI.csv")

head(df)

dfx <- df %>%

filter(measure_name == "Incidence") %>%

filter(!cause_name == "Tuberculosis") %>%

filter(location_name %in%c("Global",

"Andean Latin America", "Australasia", "Caribbean", "Central Asia", "Central Europe",

"Central Latin America", "Central Sub-Saharan Africa", "East Asia", "Eastern Europe",

"Eastern Sub-Saharan Africa", "High-income Asia Pacific", "High-income North America",

"North Africa and Middle East", "Oceania", "South Asia", "Southeast Asia",

"Southern Latin America", "Southern Sub-Saharan Africa", "Tropical Latin America",

"Western Europe", "Western Sub-Saharan Africa")) %>%

filter(sex_name == "Both") %>%

filter(age_name == "<20 years") %>%

filter(metric_name == "Rate") %>%

filter(year %in% c(1990,2021))

p <- ggplot(dfx, aes(x = val, y = location_name, fill = cause_name)) +

geom_bar(stat = "identity", position = "fill", width = 0.9) +

facet_wrap(~ year, scales = "free_y") +

labs(x = "Proportion", y = "") +

theme_classic() +

scale_x_continuous(labels = scales::percent_format()) +

scale_fill_npg(name = "") +

theme(strip.background = element_blank(),

strip.text = element_text(size = 16, face = "bold"),

axis.title.x = element_text(size = 14, face = "bold"),

axis.text.x = element_text(angle = 0, hjust = 0.5, size = 14, face = "bold"),

axis.text.y = element_text(size = 12, face = "bold"),

axis.title.y = element_text(size = 14, face = "bold"),

legend.position = "right",

legend.text =element_text(size = 14))

print(p)

ggsave(paste0("Fig3-proportion-Incidence.pdf"), width = 24, height = 12)

write.csv(dfx, paste0("Fig3-proportion-Incidence.csv"))

###############################################################################Fig4

# Merge data

file_paths <- paste0("IHME-GBD_2021_DATA-7061c5eb-", 1:7, ".zip")

data_list <- list()

for (file_path in file_paths) {

csv_file <- unzip(file_path, list = TRUE)$Name[1]

data <- read.csv(unz(file_path, csv_file))

data_list[[file_path]] <- data

}

merged_data <- do.call(rbind, data_list)

head(merged_data)

write.csv(merged_data, "204.csv", row.names = FALSE)

mapfigure1=function(GBDdf27=x,titlex="Pre",color_scheme=1){

################################################################################################

### 1. Print table at regional level

################################################################################################

df= GBDdf27 %>%

select(location_id,val) %>% left_join(.,namex)

color1 <- c("#67001f", "#b2182b", "#d6604d", "#f4a582", "#fddbc7",

"#f7f7f7", "#d1e5f0", "#92c5de", "#4393c3", "#2166ac")

# Define the base colors for each gradient

base_colors2 <- c("#457B9D", "#800080")

base_colors3 <- c("#d6604d", "#92c5de")

base_colors4 <- c("#fddbc7", "#4393c3")

base_colors5 <- c("#d1e5f0", "#FA8072")

# Create color gradients using colorRampPalette

color2 <- colorRampPalette(base_colors2)(10)

color3 <- colorRampPalette(base_colors3)(10)

color4 <- colorRampPalette(base_colors4)(10)

color5 <- colorRampPalette(base_colors5)(10)

colorx=list(color1,color2,color3,color4,color5)

colorx1=colorx[as.numeric(color_scheme)]

################################################################################################

########################

# 1.1 Incidence

########################

# Incidence

index="Incidence"

ASR=df %>% select(location_id,location,val) %>%

mutate(val=val/1)

df_asr=left_join(df_world,ASR)

xmin=min(na.omit(df_asr$val))

# Calculate the quantile breaks

breaks <- quantile(df_asr$val, probs = seq(0, 1, length.out = 11), na.rm = TRUE)

if(xmin==0){

breaks=breaks[-1]

} else{

breaks=breaks

}

print(breaks)

breaks100=1*breaks

# Initialize an empty vector to store the formatted breaks

formatted_breaks = vector("character", length(breaks100))

# Set the first break manually to start from 0

formatted_breaks[1] = paste(paste0(round(xmin,3)," -"), format(breaks100[1], digits = 4, nsmall = 1))

# Iterate over the breaks and format them

for (i in 2:length(breaks100)) {

formatted_breaks[i] = paste(paste0(format(breaks100[i - 1], digits = 4, nsmall = 1)), "-",paste0( format(breaks100[i], digits = 4, nsmall = 1)))

}

unb=unique(breaks)

if(unb[1]==xmin){

unb=unb[-1]

} else{

unb=unb

}

xmin=xmin +0.001

print(formatted_breaks)

x=cut(df_asr$val, breaks = c(xmin, unb))

print(na.omit(unique(x)))

xlen=length(na.omit(unique(x)))

xstar=length(formatted_breaks)-xlen+1

formatted_breaks=formatted_breaks[xstar:length(formatted_breaks)]

breakxxx <- quantile(df_asr$val, probs = seq(0, 1, length.out = 11), na.rm = TRUE)

values <- c(unique(breaks))

strings <- sapply(1:(length(values)-1), function(i) paste(values[i], "-", values[i+1], sep=" "))

df_asr1 <- tryCatch({

df_asr %>%

mutate(asr = replace_na(val, -99)) %>%

mutate(asr_cut = cut(asr, breaks = c(xmin, unb),

labels = formatted_breaks)) %>%

filter(!is.na(asr_cut))

}, error = function(e) {

tryCatch({

df_asr %>%

mutate(asr = replace_na(val, -99)) %>%

mutate(asr_cut = cut(asr, breaks = c(unique(breakxxx)),

labels = strings)) %>%

filter(!is.na(asr_cut))

}, error = function(e) {

message("Both attempts failed.")

NULL

})

})

ggplot(df_asr1 %>% na.omit()) +

geom_sf(aes(geometry = geometry, fill = asr_cut), size = 0.1) +

scale_fill_manual(

values = rev(colorx1[[1]]),

guide = guide_legend(reverse=T)) +

guides(fill = guide_legend(ncol = 2, title = titlex)) -> p

p1=p+ theme(axis.text.x = element_blank(),

axis.ticks.x = element_blank(),

axis.ticks.y = element_blank(),

axis.text.y = element_blank(),

legend.position = c(0.13, 0.29),

legend.background = element_blank(),

legend.key = element_blank(),

legend.title = element_text(size=8),

legend.text = element_text(size=8),

panel.grid.major = element_blank(),

panel.grid.minor = element_blank(),

panel.border = element_blank(),

panel.background = element_blank())

return(p1)

}

df <- fread("204.csv")

head(df)

dfx <- df %>%

filter(measure_name == "Incidence") %>%

filter(cause_name == "Tuberculosis") %>%

filter(sex_name == "Both") %>%

filter(age_name == "<20 years") %>%

filter(metric_name == "Number") %>%

filter(year == 2021)

titlex = "Incidence"

xa = mapfigure1(GBDdf27 = dfx, titlex = titlex, color_scheme = 1)

print(xa)

ggsave(paste0("Fig4-2021map-ASR-Incidence.pdf"), plot = xa, width = 16, height = 8)

write.csv(dfx, paste0("Fig4-2021map-ASR-Incidence.csv"))

###################################################################################3

df <- read.csv("21+1+5SDI.csv")

head(df)

dfx <- df %>%

filter(measure_name == "Deaths") %>%

filter(cause_name == "Tuberculosis") %>%

filter(location_name %in%c("Global",

"Andean Latin America", "Australasia", "Caribbean", "Central Asia", "Central Europe",

"Central Latin America", "Central Sub-Saharan Africa", "East Asia", "Eastern Europe",

"Eastern Sub-Saharan Africa", "High-income Asia Pacific", "High-income North America",

"North Africa and Middle East", "Oceania", "South Asia", "Southeast Asia",

"Southern Latin America", "Southern Sub-Saharan Africa", "Tropical Latin America",

"Western Europe", "Western Sub-Saharan Africa")) %>%

filter(sex_name == "Both") %>%

filter(age_name == "<20 years") %>%

filter(metric_name == "Rate") %>%

filter(year > 1989)

SDI21fig=function(dfx=dfx, labx="xxx"){

if (nrow(dfx) == 0) {

p1= ggplot() +

geom_text(aes(x = 0.5, y = 0.5, label = "Oops, your data is not suitable for this plot"), size = 12) +

theme_void()

spearx = "No data"

return(list(p=p1, spearx=spearx))

}

location_order <- c(

"Global", "High-income Asia Pacific", "High-income North America", "Western Europe",

"Australasia", "Andean Latin America", "Tropical Latin America", "Central Latin America",

"Southern Latin America", "Caribbean", "Central Europe", "Eastern Europe", "Central Asia",

"North Africa and Middle East", "South Asia", "Southeast Asia", "East Asia", "Oceania",

"Western Sub-Saharan Africa", "Eastern Sub-Saharan Africa", "Central Sub-Saharan Africa",

"Southern Sub-Saharan Africa"

)

colors <- c(pal_npg("nrc", alpha = 0.7)(9),

pal_aaas("default", alpha = 0.7)(9),

pal_nejm("default", alpha = 0.7)(8),

pal_jama("default", alpha = 0.7)(7))

######### 1.1 Incidence SDI 2019 in 21

DALY_2017=dfx %>% select(location_id, val, year)

df11 = left_join(DALY_2017, SDI2019)

df3=df11 %>% filter(sdi > 0.2)

df11_filtered <- df11 %>%

filter(!is.na(sdi) & !is.na(val)) %>%

mutate(location_name = factor(location_name, levels = location_order))

p1=ggplot(df11_filtered, aes(x = sdi, y = val, color = location_name, shape = location_name)) +

geom_point() +

geom_smooth(method = "loess", se = T, aes(group = 1), color = "#92A8D1") +

scale_shape_manual(values = 1:22, breaks = location_order, labels = location_order) +

scale_color_manual(values = colors, breaks = location_order, labels = location_order) +

labs(x = "SDI",

shape="", color="", x=paste0("SDI"),

y=paste0(labx," per 100,000 population")) +

theme_bw() +

theme(legend.key.size = unit(0.03,"line"),

panel.border = element_blank(),

panel.grid.major = element_blank(),

panel.grid.minor = element_blank(),

legend.background = element_blank(),

legend.title=element_text(size=12),

axis.line = element_line(colour = "black"),

legend.text=element_text(size=10)) +

guides(shape = guide_legend(nrow = 22))

# Calculate Spearman's correlation

spearman_cor <- cor.test(df11_filtered$sdi, df11_filtered$val, method = "spearman")

r <- spearman_cor$estimate; p <- spearman_cor$p.value

cat("Spearman's Correlation Coefficient (R):", r, "\n")

cat("p-value:", p, "\n")

spearx <- sprintf("r=%.4f, p=%.3e", r, p)

return(list(p=p1, spearx=spearx))

}

labx <- "ASMR"

xa = SDI21fig(dfx = dfx, labx = labx)

print(xa)

ggsave(paste0("21+1-SDI-ASR-Deaths.pdf"), plot = xa$p, width = 12, height = 8)

write.csv(dfx, paste0("21+1-SDI-ASR-Deaths.csv"))
